# Development of a Robot Arm Link System Embedded with a Three-Axis Sensor with a Simple Structure Capable of Excellent External Collision Detection

**DOI:** 10.3390/s22031222

**Published:** 2022-02-05

**Authors:** Alchan Yun, Woosub Lee, Soonkyum Kim, Jong-Ho Kim, Hyungseok Yoon

**Affiliations:** 1 Robot Center, Samsung Research, Seoul 06765, Korea; comdamdang@gmail.com (A.Y.); fieldrobot@gmail.com (W.L.); 2 Robotics and Media Institute, Korea Institute of Science and Technology (KIST), 5 Hwarang-ro 14-gil, Seongbuk-gu, Seoul 02792, Korea; kim.soonkyum@kist.re.kr; 3 Force Lab, Korea Research Institute of Standards and Science (KRISS), Daejeon 34113, Korea

**Keywords:** link embedded, strain gauge, low cost, three-axis sensor, miniaturization, collision detection

## Abstract

In order to effectively detect the contact state between the operator and the collaborative robot, a sensor with excellent external force detection performance is needed. The existing force/torque sensor and joint torque sensor, which are the two main external force sensors methods in cooperative robots, have limitations; only the force exerted at the end effector is detected, and it induces a low stiffness in the overall structure which affects the control performance. In the case of sensorless collision detection methods that utilize the current sensor that is used for motor control, the estimation of the performance of external force is sensitive to the sensor noise and dynamic model accuracy only to the extent that it can be used for collision detection. In this paper, we propose a strain gauge-based three-axis sensor of a cylindrical shape, which is often used as a link in a robot. By integrating sensors with robot links, the external force can be precisely measured without compromising the stiffness and is decoupled with joint disturbances, such as motor friction. Sensor calibration is conducted using static load evaluation equipment, and the reliability of collision detection is confirmed by comparing the theoretical/structural analysis results. Through the weight test and sensor characteristic evaluation, the performance and output stability are validated.

## 1. Introduction

Existing industrial robots do not guarantee human safety and are operated separately from humans. However, the advent of collaborative robots has resulted in the expansion and efficiency of processes by allowing people to be placed in the same production space. Accordingly, interest in technology that can ensure human safety is also increasing significantly [1,2,3]. The most fundamental element in the safe operation of a robot is a sensor that can accurately recognize the robot’s surrounding environment. Sensors for safety can be roughly divided into two categories: (1) a sensor that recognizes the surrounding environment, such as a camera or radar, that prevents the robot from colliding with an obstacle; and (2) a collision detection sensor that prevents damage by measuring the external force generated when a contact occurs. Among them, since the second type of sensor can operate reliably even in a complex surrounding environment, it can be said to be an essential element for safety [4]. 

In order to effectively detect a collision in this way, sensors using various principles are used in the field of collaborative robots. According to the measurement principle of the sensor used, it can be classified into an optical sensor type, a capacitive sensor type, a current sensor type, and a strain gauge sensor type. When the optical sensor principle is used, the change in optical distance is measured, according to the applied external force [5,6,7,8]. Palli et al. measured the deformation of the sensor frame caused by torque considering the relative position change of a LED, using the principle of detecting the change in the photocurrent flowing through the photodetector [6]. Al-Mai et al. detected an external force corresponding to six axes, as a result of applying a reflection intensity control and a new correction technique using six optical fibers [7]. Shams et al. measured the torque generated by measuring the angular displacement in manufactured gear and a photo-interrupter [8]. 

When the capacitive sensor principle is used, a collision is detected using the change in capacitance generated between electrodes due to an external force [9,10,11,12,13,14,15]. Kim et al. simplified the complex structure and assembly process of existing sensors, and effectively manufactured a sensor. In addition, by applying a fringe effect considering non-linearity, the manufactured sensor had high sensitivity [10]. Kim et al. fabricated a sensor filled with an elastomer in a plastic sensor structure, measured the change in capacitance caused by deformation of the sensor, and estimated the external force using the change of the capacitance [11]. Kim et al. calculated the input torque by measuring the change in the distance between the electrodes using the capacitive principle. By using a flexural structure, the sensor had a wide measurement range while maintaining high rigidity [13]. In the case of using the current sensor principle, an external collision is determined using the change in current generated by an external force, such that no additional sensor is required [16,17,18,19]. Je et al., by adjusting the current value and the input value of the controller, detected collisions while minimizing the damage [16]. Chen et al. detected collisions in real-time by measuring the motor current and position information from the encoder of a robot joint [18]. However, performance changes occur depending on the robot’s dynamic model accuracy and the control algorithm used. In addition, it is difficult to know the exact external force, for this is greatly affected by the frictional force compensation performance, which is the most problematic factor in terms of robot control. Strain gauge sensors are the most used, due to their ease of access and low price, compared with other sensors. Torque is measured by converting a small amount of deformation caused by an external force into an electrical signal, such that collisions with the external environment can be detected using this principle [20,21,22,23,24,25,26,27,28]. According to the number of strain gauge sensors applied to the robot arm and their attachment positions, it is possible to detect a collision or reduce manufacturing cost, thereby controlling the sensing performance of external force. Jung et al. measured stable torque values in various speed environments by attaching a sensor to the flexspline of the harmonic drive and using a torque ripple filtering algorithm based on order tracking analysis [24]. Min et al. manufactured a torque sensor with improved rigidity while minimizing the influence of the harmonic drive. In addition, the volume of the existing joint module does not change, making it easy to replace the existing sensor [26]. 

Among the sensors with the above various measurement principles, according to the location where the strain gauge sensor is installed, it can be divided into a joint-torque sensor and a force/torque sensor as follows. In the case of a joint-torque sensor, it is located in the joint part of each link, and collision detection is possible in all links. In general, a spoke type structure that undergoes large deformation is manufactured, a strain gauge sensor is attached, and the torque generated by an external force is measured [20,23,24,25,26,27]. Aghili et al. manufactured a sensor which significantly improved the torsional rigidity, compared to conventional sensors, by attaching a strain gauge to a hexagonal torque sensor optimized through structural analysis [20]. Kim et al. had the advantage of using CNTs to simplify the fabrication and attachment of sensors, but noted limitations in material properties with poor linearity [23]. Ubeda et al. conducted structural optimization in spoke-type structures of various shapes through finite element analysis in order to determine the greatest deformation. The performance of the sensor was evaluated by attaching a strain gauge to the place where the greatest deformation occurred [25].

As it is applied to all joints, the price increases, and the stiffness of the sensor is often sacrificed to cause large deformation. For the detection of collisions with workers who share the same work environment, there is a lack of performance evaluation of the sensor for micro external force [20,23,24]. In the case of a force/torque sensor, it has been used for a long time for interaction with a robot and has the most accurate external force measurement reliability. It is mainly attached to the end-effector of the robot arm, measures the force and moment, corresponding to six axes to estimate the external force, and has the disadvantage that the sensor is expensive [22,28,29]. Sun et al. fabricated a sensor using 32 strain gauges in order to compensate for the coupling errors caused by different axial external forces [22]. Kim et al. used 16 strain gauge sensors for force/moment calculation and decoupling of interfering external forces. Furthermore, the stability of the sensor structure was verified through structural analysis [28]. Kim et al. used structural analysis to manufacture a sensor for efficient force/moment calculation, and attached 20 strain gauges to satisfy the interference error at 3% of that of a commercial sensor [29]. In this way, many studies using strain gauges have been conducted to effectively detect external forces, but there are still many factors to consider in terms of the location of the sensor and the effect of the number of sensors used.

When a strain gauge is used in a robot arm for external force extraction, there may be problems in terms of manufacturing a structure for attaching the sensor and durability/reliability, according to the number of sensors. First, in the case of joint-torque sensors, the sensor is attached to the manufactured spoke-type structure, which is additionally mounted to the joint [20,23,24,25,26,27]. At this time, in the process of fabricating the structure and attaching the sensor, relatively high time and costs are required for structural stability, the improvement of sensing performance, and reduction of manufacturing cost. In order to effectively reduce the time and cost consumed in this process, various approaches are required. Second, in the case of studies using strain gauges, a relatively large number of sensors have been used to improve the external force sensing performance and minimize the interference error with respect to other axial directions [22,28,29]. As a result, in the process of attaching the sensor and connecting the signal line, the structure of the sensor system becomes complicated and the manufacturing difficulty, time, and cost increase. In addition, as the number of sensors increases, the probability of the occurrence of defects increases, such that it becomes difficult to obtain relatively good sensor stability and durability/reliability.

**Figure 1:**
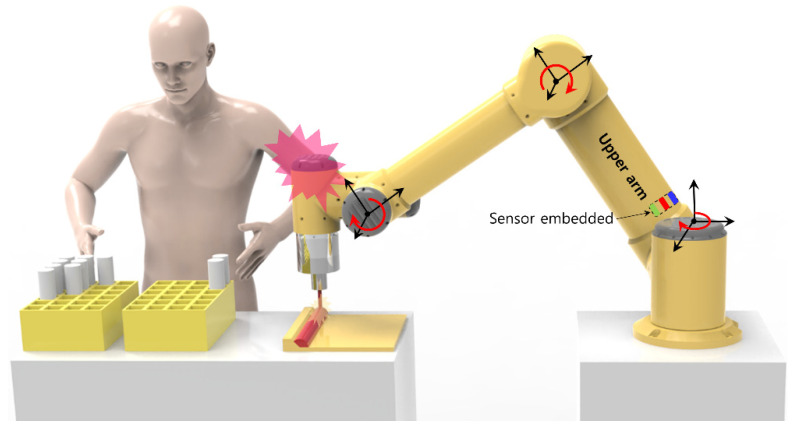
A robot arm link embedded with a three-axis sensor detects a collision with an operator.

Therefore, in this paper, to overcome the limitations of current sensor and strain gauge sensor studies applied to the existing robot arm, we propose a robotic arm system in which: (1) the structure is simple and inexpensive, (2) there is no sacrifice of the rigidity of the robot arm, and (3) there is excellent collision detection. In particular, it was intended to be applied to the work in which a moment due to a compressive load is minimized, such as handling light parts or simple repetitive tasks, such as welding (See Figure 1). The proposed system has the following features. First, versatility was secured by embedding three full Wheatstone bridges on the surface of a cylindrical shape object used as a link in many robots. Second, it is possible to acquire data without an additional structure by attaching a sensor to the surface of the link. In the case of a joint-torque sensor installed in a joint, there is an effect of lowering the rigidity, which tends to cause a problem in terms of control precision. However, in this study, by attaching a small number of sensors to the surface of the link, there is an advantage in that the external force can be estimated without deterioration of durability, reliability and rigidity. Also, in the case of a general force/torque sensor, there is a limitation in that only the external force applied to the end effector can be measured, but in this study, the external force applied to the link can also be measured. Accordingly, a sensor system was built with a very low manufacturing cost and a short manufacturing period, with which excellent collision detection precision could be achieved, through the use of the strain-gauge sensor. Third, in order to effectively detect the contact state with surrounding workers, the micro external force extraction and collision detection performance were evaluated, using a weight and the robot base axis. Fourth, the possibility of detecting an external force in a dynamic environment was evaluated, by adjusting the speed at which the link slope changes. Through structural analysis, the rigidity of the link was considered, and the reliability of the calibration moment was confirmed. In addition, the durability and reliability of the sensor were verified by evaluating its hysteresis, repeatability, and drift characteristics.

## 2. Development of the Three-Axis Sensor Embedded in the Link

### 2.1. External Force Estimation Principle

**Figure 2:**
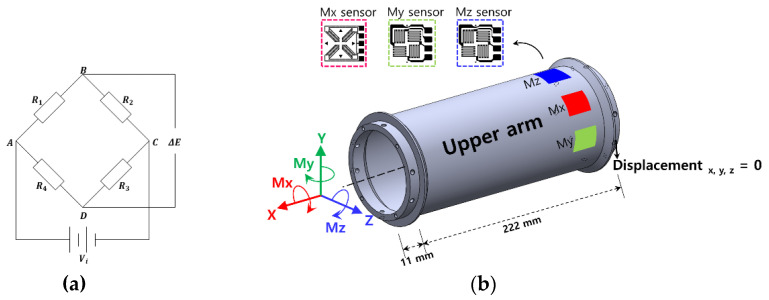
Collision detection system configuration using Wheatstone bridge; (**a**) Wheatstone bridge circuit used for measurements; (**b**) attach a strain gauge to the link surface to collision detection.

To estimate the external force in this paper, as shown in Figure 2a, a Wheatstone bridge circuit was constructed. By using a full bridge circuit structure, it is possible to obtain a stable sensor signal, where the resistance of the sensor can be changed by an external force. As shown in Figure 2b, when a bending moment is generated by an external payload in a link with one end fixed, deformation occurs in the strain gauge attached to the link. At this time, in the Wheatstone bridge attached to the link, the sensor is deformed due to the bending moment (Mz), compressive deformation is generated in *R*_1_ and *R*_3_, and tensile deformation is generated in *R*_2_ and *R*_4_. The change in resistance is expressed by Equations (1) and (2), as follows:

(1) $$\frac{\Delta R_{1}}{R_{1}}=\frac{\Delta R_{3}}{R_{3}}=k\varepsilon_{xx},$$

(2) $$\frac{\Delta R_{2}}{R_{2}}=\frac{\Delta R_{4}}{R_{4}}=k\varepsilon_{yy},$$

(3) $$\upsilon =-\frac{\varepsilon_{yy}}{\varepsilon_{xx}},\;$$

where *ε* is the strain in each direction and *υ* denotes Poisson’s ratio, as expressed in Equation (3). The bending moment can be calculated using the change of strain in the Wheatstone bridge circuit given in Equations (1)–(3), as shown in Equation (4):

(4) $$\frac{\Delta E}{V_{i}}=\frac{1}{4}\left(\frac{\Delta R_{1}}{R_{1}}-\frac{\Delta R_{2}}{R_{2}}+\frac{\Delta R_{3}}{R_{3}}-\frac{\Delta R_{4}}{R_{4}}\right)=\frac{1}{4}k\left(\varepsilon_{1}-\varepsilon_{2}+\varepsilon_{3}-\varepsilon_{4}\right)=\frac{1}{2}k\varepsilon_{xx}\left(1+\upsilon \right)$$

where Δ*E* is the output voltage, *V_i_* is the input voltage, Δ*R/R* is the resistance change for each sensor, and *k* is the gauge factor, which is the same for all four sensors. When a torsional moment occurs in the axial direction of the link, as in Figure 2b, the strain is measured by the strain gauge attached to the Mx sensor part of the link. At this time, deformation occurs due to the torsional moment (Mx) in the Wheatstone bridge, that is, the Mx sensor. *R*_1_ and *R*_3_ are compressive strains, while *R*_2_ and *R*_4_ are tensile strains, which can be calculated as follows:

(5) $$\frac{\Delta E}{V_{i}}=\frac{1}{4}\left(\frac{\Delta R_{1}}{R_{1}}-\frac{\Delta R_{2}}{R_{2}}+\frac{\Delta R_{3}}{R_{3}}-\frac{\Delta R_{4}}{R_{4}}\right)=\frac{1}{4}k\left(\varepsilon +\varepsilon +\varepsilon +\varepsilon \right)=k\varepsilon \;$$

Through Equations (4) and (5), the full bridge output voltage can be obtained, using the sensor deformation. To estimate the external force, the voltage data obtained by the deformation of the sensor and the structural analysis result are used. Using the structural analysis results obtained under the same conditions, calibration is performed, and the external force can be estimated from the output voltage obtained from the strain gauge.

### 2.2. Manufacture of the Three-Axis Sensor and Setup of the Measurement System

**Figure 3:**
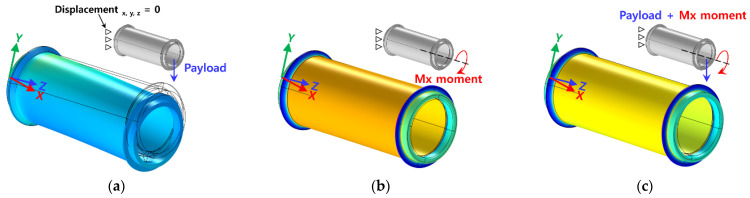
Link dimension selection using structural analysis. (**a**) Stress distribution of link when payload (10 kg, Mz moment) is applied (principle stress: 5.30 MPa, principle strain: 6.27 × 10^−5^). (**b**) Stress distribution of link when Mx moment (300 Nm) is applied (principle stress: 15.15 MPa, principle strain: 2.92 × 10^−4^). (**c**) Stress distribution of link when the payload (10 kg, Mz moment) and Mx moment (300 Nm) are applied simultaneously (principle stress: 18.19 MPa, principle strain: 3.11 × 10^−4^).

In this study, the goal was to develop a sensor system with excellent collision detection performance with a simple structure, low cost, and short manufacturing period. Structural analysis was performed using a commercial analysis program (COMSOL Multiphysics 5.5, Altsoft), in order to select the specifications of the cylindrical link. The specifications of the link, shown in Figure 2b, were selected considering the deformation efficiency. Considering the structural stability and sensitivity of the sensor, the total length was 244 mm and the thickness was 3 mm. Structural analysis was performed to confirm the structural stability of the selected specifications. A boundary condition was set, in the form of completely fixing one end of the link, while the link’s own weight (0.7 kg) was considered as the initial load condition. Considering the operating environment of the robot arm link, a load of 10 kg (Mz moment) and a Mx moment of 300 Nm were considered as the maximum payload. Figure 3a,b show that, when a load of 10 kg (Mz moment) and a Mx moment of 300 Nm were considered, the maximum principal stress (maximum principal strain) occurred at 5.30 MPa (6.27 × 10^−5^) and 15.15 MPa (2.92 × 10^−4^), respectively. Compared to Al 6061-T6 (yield stress: 240 MPa), which was used as the material of the link, less than 10% stress occurred, satisfying the material property stability requirement. To check the structural stability under various payload conditions in the robot arm, a load of 10 kg (Mz moment) and a Mx moment of 300 Nm were simultaneously considered. The results are shown in Figure 3c: the maximum principal stress (maximum principal strain) was 18.19 MPa (3.11 × 10^−4^). Allowable stress occurred even under complex load conditions, confirming the structural stability of the selected specifications.

As the resistance due to micro-deformation needs to be measured, a full bridge circuit with excellent sensitivity and temperature compensation was selected to configure the sensor system. The information of the used strain gauge sensor is as follows. The strain gauge for the moment was an N2K-S5193R-350/E5 (Micro-Measurement Co., Wendell, NC, USA), which has 1.80 mm gauge length, two-gauge factor, and 350 ohms resistance. The strain gauge for torque was an N2K-S5023M-10C/DG/E3 (Micro-Measurement Co., Wendell, NC, USA), which has 1.15 mm gauge length, two-gauge factor, and 1000 ohms resistance. In order to obtain the exact position of the sensor to be attached in the axial direction of the link, the structural analysis result was used. As a result, three strain gauge sensors were attached 38 mm away from the left end, where the stress was the highest and evenly distributed. To measure the My, Mz bending moment, one strain gauge was attached to the top surface of the link, while another was attached to the surface rotated by 90 degrees, in order to prevent interference. The strain gauge for measuring the Mx torsional moment was located between the My and Mz strain gauges (i.e., rotated by ±45 degrees in the axial direction), such that the largest deformation due to external forces occurred.

**Figure 4:**
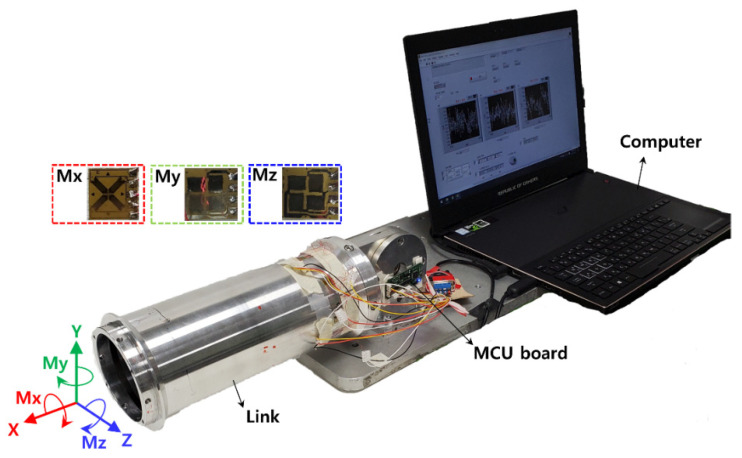
Experimental setup for calibration and performance.

The sensor data was calibrated and the noise was minimized using a Micro Controller Unit (MCU) board (sampling rate, 30 Hz) for primary signal processing, which was attached to the link. A change in resistance caused by an external force was converted into an analog voltage value, using an input power of 5 V. The link specification, as verified through structural analysis, satisfied the sensor’s output voltage range (−19~19 mV). It was converted into a digital value with a resolution of 2.264 × 10^−6^ mV, using an Analog–Digital-Converter (ADC) chip (ADS1231, 24-bit, Texas Instruments., Dallas, TX, USA). The converted data was connected to a program installed on a laptop (LabVIEW 2018, National Instruments Corp., Austin, TX, USA) using a Serial Peripheral Interface (SPI) for communication, such that the external force could be estimated. Figure 4 shows the signal processing board and PC connected to the robot arm link, with three full bridge circuits embedded. Through structural analysis, the robot arm link was manufactured and the optimal sensor attachment position was obtained. The sensor data could be checked on the laptop, through signal processing and SPI communications. As a result, a robot arm link with an embedded three-axis sensor was manufactured, such that we could construct a system which is capable of estimating external forces using the measured sensor data.

## 3. Calibration of the Three-Axis Sensor

### 3.1. Fabrication of Static Load Evaluation Equipment

**Figure 5:**
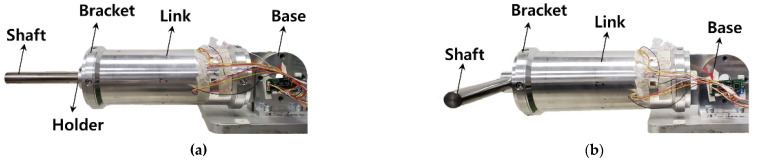

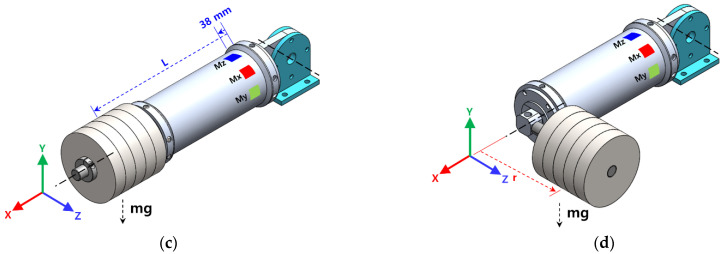
Link system coupled to static load evaluation equipment. (**a**) Mechanical part for applying bending moment (My, Mz). (**b**) Mechanical part for applying torsional moment (Mx). (**c**) Link system affected by Mz Moment by mg. (**d**) Link system affected by Mx and Mz moment by mg.

A sensor evaluation device capable of applying a static load was manufactured, where the external force could be estimated with the strain gauge sensor system embedded in the link. As shown in Figure 5a, it consisted of a base fixing the combined link, a bracket (Al6061-T6), a shaft (S45C) where the weight (SM15C) is supported, and a holder (Al200) fixing the position of the weight. The base joined to the right side of the link was fixed to the table and, as shown in Figure 5c, a Mz moment was generated by applying weight to the shaft attached to the left side of the link. At this time, the generated sensor data were acquired from the Mz sensor. In the same way, if the link was rotated 90 degrees and weight is applied, the My moment sensor data were acquired from the My sensor. As shown in Figure 5b,d, a torsional moment was generated by applying a weight to the bracket attached to the link in a clockwise direction. At this time, the Mx and Mz moment data, simultaneously generated by the mechanical part, were acquired from the Mx and Mz sensors. From 0 kg to 10 kg, weight was applied at 2 kg intervals; meanwhile, from 0 to 90 degrees (at 10 degrees intervals), the link slope was adjusted. The link had a self-weight of about 0.7 kg. In the absence of weight, as in the case of Figure 5a, it had an initial weight of about 1.4 kg. Figure 5b had an initial weight of about 1.45 kg.

### 3.2. Sensor Calibration

**Figure 6:**
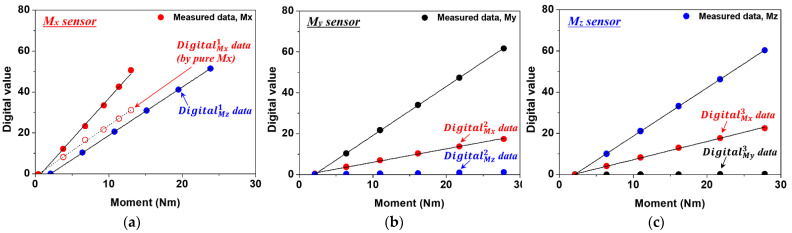
Calibration curve of three-axis sensor obtained through static load evaluation equipment. (**a**) Sensor data measured by *Mx* sensor. (**b**) Sensor data measured by *My* sensor. (**c**) Sensor data measured by *Mz* sensor.

As in Figure 5a,b, the measurement system was configured to obtain the calibration curve of the proposed sensor system, using static load evaluation equipment. Using 2 kg weights, loads from 0 to 10 kg were applied and reduced. The accuracy of the measurement results was improved by using the average value of three repetitions. As shown in Figure 5c,d, 3D geometric modeling and structural analysis were performed under the same conditions as the sensor measurement environment. The moment change caused by the load was calculated based on the sensor position. In addition, in order to verify the reliability of the structural analysis result, the moment generated by the weight was calculated through theoretical calculation, as follows:

(6) $$M_{x}=m\cdot g\cdot r$$

(7) $$M_{y},\;M_{z}=m\cdot g\cdot L$$

where *m* is the weight, *g* is the acceleration due to gravity (9.81 m/s^2^), and *r* and *L* are the center of gravity distances for calculating each moment, calculated as 114.22 mm and 250.25 mm, respectively. Figure 6a–c show the calibration curve results of sensor data measured under weights ranging from 0 to 10 kg.

The data measured by each sensor can be expressed as follows:

(8) $$Digital_{MX}^{T}=Digital_{MX}^{1}+Digital_{MX}^{2}+Digital_{MX}^{3}$$

(9) $$Digital_{MY}^{T}=Digital_{MY}^{1}+Digital_{MY}^{2}+Digital_{MY}^{3}$$

(10) $$Digital_{MZ}^{T}=Digital_{MZ}^{1}+Digital_{MZ}^{2}+Digital_{MZ}^{3}$$

$Digital_{MX}^{T}$ means the sum of Mx sensor data measured by three sensors, and $Digital_{MY}^{T}$, $Digital_{MZ}^{T}$ mean the sum of My and Mz sensor data, respectively. $Digital_{MX}^{1}$ means Mx data measured by the Mx sensor, and $Digital_{MY}^{1}$ and $Digital_{MZ}^{1}$ mean My data and Mz data measured respectively. $Digital_{MX}^{2}$ and $Digital_{MX}^{3}$ mean Mx data measured by My and Mz sensors, respectively. Under the influence of evaluation equipment, the Mx and Mz moments occur simultaneously. As a result, in Figure 6a, Mx sensor data and Mz sensor data are measured simultaneously. Mx sensor data measured by Mx and Mz moments applied at the same time can be said to be sensor data in which the effects of Mx and Mz moments are mixed. To make sensor data mixed by two moments into sensor data by one moment, the data was processed as follows. In order to acquire pure Mx sensor data generated by Mx moment from the Mx sensor in Figure 6a, Mx sensor data generated by Mz moment in Figure 6c was used. In Figure 6b,c, $Digital_{MX}^{2}$ by My moment and $Digital_{MX}^{3}$ by Mz moment were simultaneously obtained by the Mx sensor attached to the side of the My and Mz sensors at 45 degrees. The Mz moment generated in Figure 6a is applied to the calibration curve obtained from the Mx sensor of Figure 6c to obtain Mx sensor data by Mz moment. Finally, if the mixed Mx sensor data in Figure 6a is removed with the Mx sensor data obtained in Figure 6c, pure Mx sensor data and the calibration curve by the Mx moment is obtained. As a result, the pure Mx sensor data, $Digital_{MX}^{1}$ is obtained from the mixed Mx sensor data.

In the case of $Digital_{MY}^{1}$ obtained in Figure 5d, considering the relatively weak sensor signal and corresponding moment, it was assumed to be 0. $Digital_{MZ}^{2}$ and $Digital_{MY}^{3}$ obtained in Figure 6b,c, were also very weak sensor signals compared to $Digital_{MY}^{2}$ and $Digital_{MZ}^{3}$, and were assumed to be 0. The data measured by each sensor can be expressed as follows:

(11) $$Digital_{MX}^{1}=2.4876*M_{MX}-1.19752$$

(12) $$Digital_{MY}^{1}=0$$

(13) $$Digital_{MZ}^{1}=2.3609*M_{MX}-4.85058$$

(14) $$Digital_{MX}^{2}=-0.66033*M_{MY}+0.5905$$

(15) $$Digital_{MY}^{2}=2.39987*M_{MY}-4.88184$$

(16) $$Digital_{MZ}^{2}=0$$

(17) $$Digital_{MX}^{3}=0.88596\;*M_{MZ}-1.65198$$

(18) $$Digital_{MY}^{3}=0$$

(19) $$Digital_{MZ}^{3}=2.34507\;*M_{MZ}-4.81393$$

In the case of $Digital_{MX}^{2}$ obtained in Figure 5d, it has a -slope, but it is expressed in reverse for comparison with other curves. Using the above equations, it is represented by the matrix in Equation (20):

(20) $$\left[\begin{array}{c} Digital_{MX}^{T}\\ Digital_{MY}^{T}\\ Digital_{MZ}^{T} \end{array}\right]=\left[\begin{array}{ccc} 2.4876 & -0.66033 & 0.88596\\ 0 & 2.39987 & 0\\ 0 & 0 & 2.34507 \end{array}\right]\left[\begin{array}{c} MX\\ MY\\ MZ \end{array}\right]+\left[\begin{array}{c} -2.259\;\\ -4.88184\\ -4.81393 \end{array}\right]$$

The moment calculated by each sensor is organized as follows using matrix in Equation (21).

(21) $$\left[\begin{array}{c} MX\\ MY\\ MZ \end{array}\right]={\left[\begin{array}{ccc} 2.4876 & -0.66033 & 0.88596\\ 0 & 2.39987 & 0\\ 0 & 0 & 2.34507 \end{array}\right]}^{-1}(\left[\begin{array}{c} Digital_{MX}^{T}\\ Digital_{MY}^{T}\\ Digital_{MZ}^{T} \end{array}\right]-\left[\begin{array}{c} -2.259\;\\ -4.88184\\ -4.81393 \end{array}\right])$$

To confirm the reliability of the moment shown in Equation (21), the calculation results using Equations (6) and (7) and the structural analysis results were compared. Structural analysis results were considered as a standard, and results of 13.02 Nm(Mx), 27.82 Nm(My), and 27.82 Nm(Mz) were obtained, respectively. When a 10 kg load was applied, a moment deviation of about 1.5% (0.19 Nm) was generated in the Mx sensor, and about 0.5% (0.16 Nm) in the My and Mz sensors. The reliability of the analysis results was confirmed through comparison, and the moment was estimated using the sensor data measured when a 10 kg load was applied along with the matrix in Equation (21). Moments of 13.25 Nm, 27.74 Nm, and 27.75 Nm were obtained in the Mx, My, and Mz sensors, where errors of about 1.76%, 0.27%, and 0.25% occurred, compared to the analysis results. As a result, excellent external force estimation results were obtained by decoupling the mixed sensor data by Mx and Mz moments.

**Figure 7:**
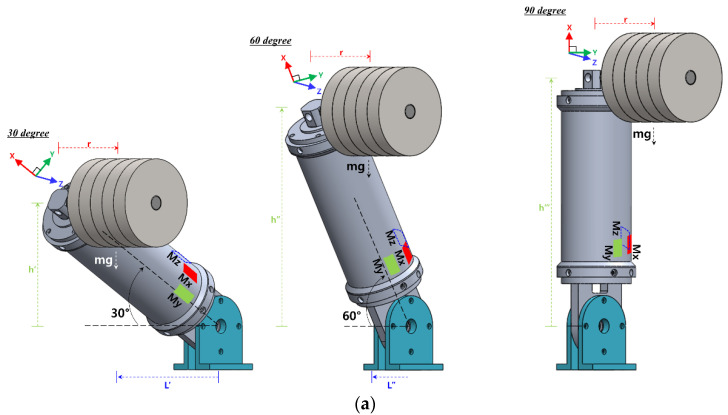

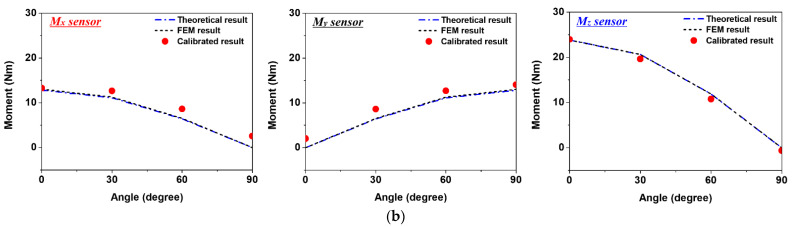
External force estimation result of three-axis sensor considering the slope of link. (**a**) Schematics of experiments. (**b**) External force estimation result considering the slope of the link.

As shown in Figure 5d, when torsional and bending moments occur simultaneously, the moment estimated from each sensor, according to the slope of the link, was compared with the theoretical calculation formula and the structural analysis result. Equations (6) and (7) were used to calculate the moment and, as the slope of the link increased, the distance from the center of gravity decreased. The applied load was considered to be 10 kg, and sensor data were acquired (at intervals of 30 degrees) from 0 degrees to 90 degrees in the horizontal state. The measured sensor data were calculated as a moment for each axis using Equation (21). Figure 7a shows the schematic diagrams for geometric modeling at 30 degrees, 60 degrees, and 90 degrees (thus excluding 0 degrees), and Figure 7b shows the moment calculated by each sensor. Before comparing the moment obtained from the sensor, we confirmed that the moment calculated through the theoretical calculation formula and the structural analysis result agreed well. 

For the Mx and Mz moment, shown in Figure 7b, the estimation error was larger in the vertical state than in the horizontal state of the link slope. This is because when the link was in a vertical state, the Mx and Mz moments have the smallest occurrence. In the case of the Mx sensor, as the slope of the link increase, the error in estimating the external force increases due to the influence of the decreasing moment. Except for the vertical state (i.e., 90 degrees), when the slope of the link is 60 degrees, the maximum external force estimation error occurs; the moment difference at this time was 2.09 Nm.

When in the horizontal state, the largest external force estimation error occurred in the My sensor that is shown in Figure 7b. 

This is because, due to the influence of the relatively short center of gravity distance, the moment change due to the link slope was the smallest. As a result, as the link slope changes from the horizontal state to the vertical state, the error with the analysis result decreases. So, as the link slope moved from 60 degrees to vertical (i.e., 90 degrees), the moment estimation error decreased from 12.68% to 7.83% compared to the analysis result. In the case of the Mz sensor, the external force estimation error occurs the least compared to other axis sensors under all conditions except for the vertical state. When the slope of the link is 60 degrees, the maximum external force estimation error is 1.14 Nm, which is about 9.56% of the analysis result.

In the current evaluation equipment, the force and moment occurred simultaneously by weight. Depending on the slope of the link, the force remained constant, while the moment changed, resulting in estimation error. As a result, when the influence of the moment is minimized, the sensor has the largest external force estimation error. To address this problem, Kim et al., decoupled the force and moment by additionally placing a strain gauge on the same straight line of the existing sensor [28]. However, the sensor arrangement proposed in this study can only calculate the moment corresponding to the three axes using the minimum number of sensors.

### 3.3. Evaluation of Sensor Characteristics

**Figure 8:**
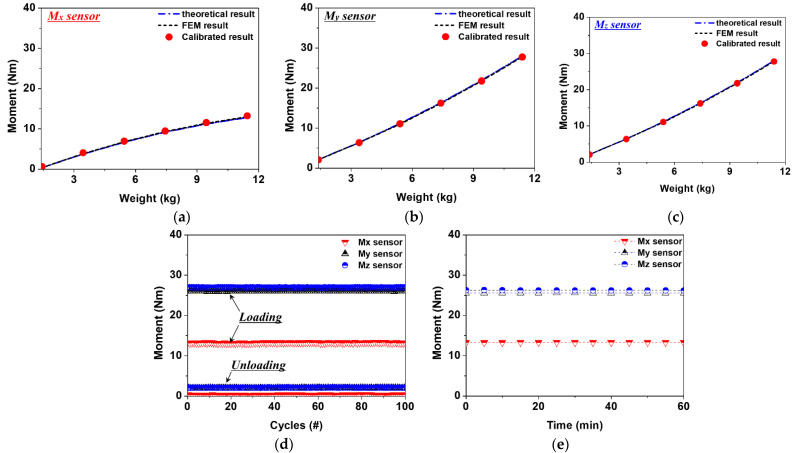
The three-axis sensor according to weight change and evaluation of sensor characteristics: (**a**) hysteresis result of Mx sensor; (**b**) hysteresis result of My sensor; (**c**) hysteresis result of Mz sensor; (**d**) evaluation of the repeatability; (**e**) evaluation of the drift characteristics.

To check the characteristics and durability/reliability of the proposed three-axis sensor embedded in the link shown in Figure 4, the hysteresis, repeatability, and drift characteristics of the measurement were evaluated. In Figure 8a–c, the sensor data, measured according to the increase and decrease of the load, are presented as calibration moments. It was confirmed that the moment calculated by the sensor fits well with the structural analysis and theoretical calculation results. The My and Mz sensors used to measure the bending moment had hysteresis errors of 0.17% and 0.26%, respectively. For the Mx sensor, used to measure the torsional moment, a hysteresis error of 0.13% occurred. We confirmed that similar or superior sensor characteristics were satisfied, compared to previous research results [15,21,25,26,28]. Figure 8d shows the repeatability characteristics of each sensor. A maximum load of 10 kg was repeatedly applied for 100 cycles, and the measured sensor data were calculated as a moment. The Mx, My, and Mz sensors showed almost constant results for 100 repeated load cycles, and the repeated durability characteristics of 0.5%, 0.33%, and 0.22% could be confirmed, respectively. It can be seen that, even if a high load was repeatedly applied to the sensor, stable output characteristics were observed. In order to confirm the change in the output characteristics of the sensor over time, as shown in Figure 8e, a 10 kg load was continuously applied for 60 min. Sensor data were measured at 5 min intervals and calculated as moments. The drift errors of the Mx, My, and Mz sensors were 0.19%, 0.17%, and 0.08%, and were calculated as moments of 0.03 Nm, 0.06 Nm, and 0.12 Nm, respectively. It was confirmed that, even when a high load was continuously applied, the external force could be reliably detected from the robot arm link, due to the sensor output characteristics.

## 4. Evaluation of Detection Performance

### 4.1. Evaluation of the Sensor’s Micro Force Detection Performance through Weight Test

**Figure 9:**
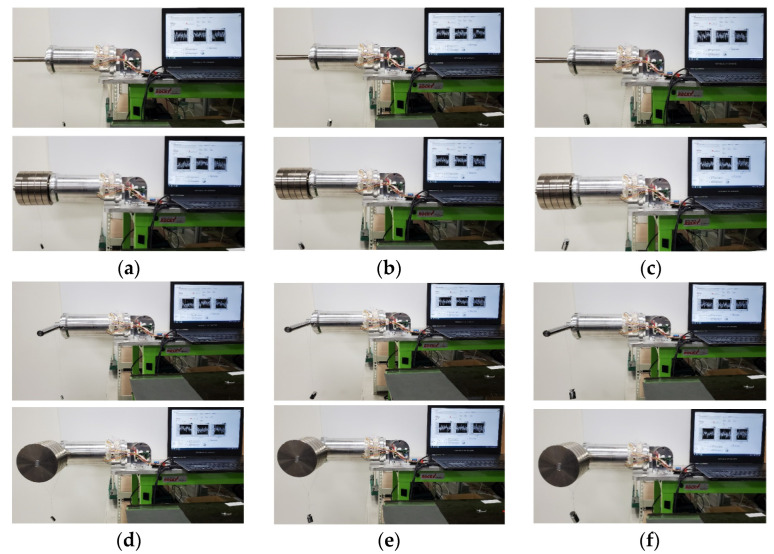
Weight (10 g, 50 g, 100 g) test with or without initial weight (10 kg): (**a**) bending moment by 10 g; (**b**) bending moment by 50 g; (**c**) bending moment by 100 g; (**d**) torsional moment by 10 g; (**e**) torsional moment by 50 g; (**f**) torsional moment by 100 g.

**Figure 10:**
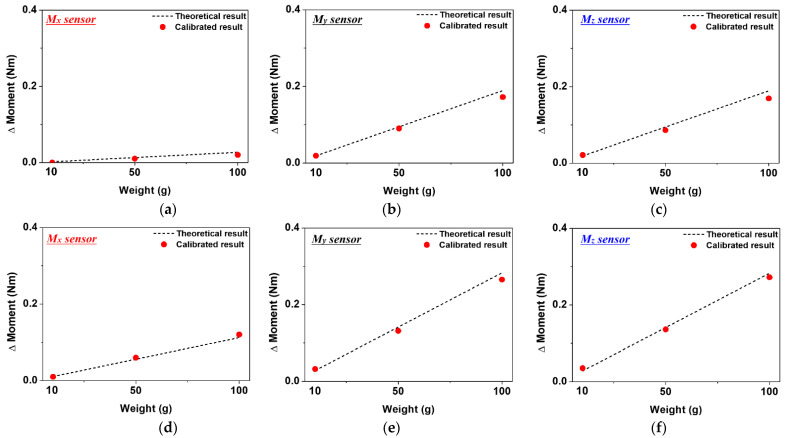
The evaluation result of micro external force detection performance of three-axis sensor by weight: (**a**) the measurement result of the Mx sensor without initial weight; (**b**) the measurement result of the My sensor without initial weight; (**c**) the measurement result of the Mz sensor without initial weight; (**d**) the measurement result of the Mx sensor under 10 kg weight; (**e**) the measurement result of the My sensor under 10 kg weight; (**f**) the measurement result of the Mz sensor under 10 kg weight.

In order to evaluate the collision detection performance of the link embedded with the three-axis sensor, the following experiment was conducted (see Figure 9). In order to check the detection performance according to the initial load, the measurement was carried out by dividing the condition into a no-load condition and when a load of 10 kg was applied. The data generated by the Mx, My, and Mz sensors were measured with 10 g, 50 g, and 100 g weights. A weight was applied to the center of gravity, in order to cause the moment to change, and a situation in which a micro external force was applied to the link was simulated. The sensor data generated by the initial load condition of the link and the weight were converted into a moment, using the matrix shown in Equation (21) obtained through the previous experiment. The sensor’s micro external force-sensing performance was evaluated by comparing the moment obtained using the theoretical calculation formula and the moment using a calibration curve under the same conditions. In Figure 10a,d, the change in the torsional moment generated with 10 g, 50 g, and 100 g weights under the no-load and the 10 kg pre-load condition of the Mx sensor is compared with the theoretical calculation results.

In Figure 10a, the change in torsional moment due to the weight of 10 g was not detected, but was detected with the 50 g weight. At this time, the generated moment was calculated as 0.01 Nm, and an error of about 23% occurred in the theoretical calculation result. In Figure 10d, using an initial load of 10 kg, the torsional moment generated with the same weight, as a whole, increased. The sensing performance increased under the influence of the initial load, and it was possible to detect a change in the moment due to a 10 g weight. At this time, the generated moment was calculated as 0.01 Nm, which causes an error of about 9% which occurred in the theoretical calculation result, and the minimum torque that can be measured by the Mx sensor can be inferred. Figure 10b,e show the change in the moment generated with weight of the My sensor under no load and 10 kg pre-load conditions, respectively. Under both load conditions, the external force generated by the minimum weight of 10 g was detected, and the generated moment was calculated to be about 0.02 Nm and 0.03 Nm. Figure 10c,f show the moment change and theoretical calculation results under each load condition measured by the Mz sensor. Like the My sensor, for weights of 10 g, 50 g, and 100 g, external forces were all detected, and changes by 10 g in the external force were detected, even in the absence of a 10 kg pre-load. The moment generated at this time was calculated as 0.02 Nm and, as the magnitude of the generated moment increased, the theoretical calculation result error tended to decrease. Through the experiment, it was confirmed that the proposed three-axis sensor system accurately detected changes in the micro external force, enabling excellent collision detection.

### 4.2. Evaluation of the Sensor Tilt Detection Performance Using the Robot Base Axis

**Figure 11:**
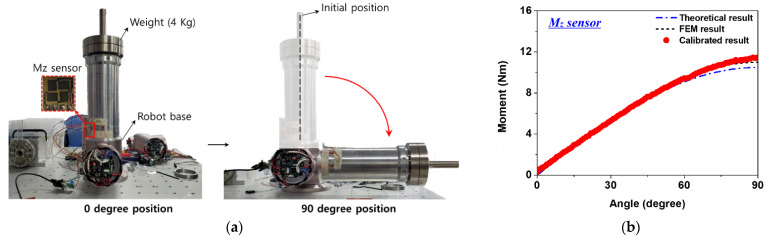
Slope detection performance evaluation result of a link embedded with three-axis sensor: (**a**) the link connected to the robot base axis; (**b**) moment estimation result of Mz sensor according to link slope change.

To check the detection performance according to the change in the slope of the link embedded with the sensor, as shown in Figure 11a, the link was coupled to the base axis of the cooperative robot. The base axis of the robot used for link coupling was composed of a harmonic drive, a BLDC motor, an absolute encoder, and a brake. In this experiment, it moved in the direction of the one degree of freedom. A total weight of 5.4 kg, including a load of 4 kg, was considered, and the slope of the link was changed from 0 degrees (vertical state) to 90 degrees (horizontal state) at intervals of 1 degree. The distance to the center of mass moved according to change the slope of the link, and the Mz sensor data were measured at that time.

Figure 11b shows the theoretical calculation and structural analysis results, according to the link slope change, and the moment calculated by the calibration curve. As the slope of the link approached the horizontal state, the stress increased and a stress concentration was generated. Therefore, in the future, when the link is applied to the robot arm, assuming a constant weight and posture, the stress distribution can be predicted. Structural analysis was performed in the same way as for the experimental conditions, and a calibration curve (y = 2.8869x − 0.8992) was obtained using the sensor data. Assuming the structural analysis result as reference data, it was compared with the external force estimation result calculated through sensor calibration. Before comparison, the reliability of the structural analysis results was obtained using the theoretical calculation results. Calculation results using the calibration curve were obtained at intervals of 1 degree, showing almost the same trend as the structural analysis results. The graph showed a linear behavior and, as the slope of the link increased, it became a sine wave. From 0 to 10 degrees, when the slope of the link changed by 1 degree, the average moment changed by about 0.17 Nm. From 11 to 90 degrees link slopes, the moment had an error of less than 2.4%, on average, compared to the structural analysis result. When the slope changed by 1 degree in this section, an average moment changes of about 0.11 Nm occurred. It was possible to determine the level of external force detection that could be estimated according to the change in the link slope, and the reliability could be confirmed through comparison with the structural analysis results.

### 4.3. Evaluation of the Sensor’s Dynamic State Detection Performance, According to the Link Slope Speed Change

**Figure 12:**
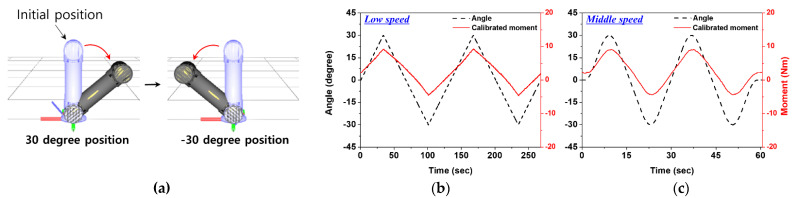
Evaluation of sensor’s external force detection performance according to the link’s slope change speed: (**a**) range of slope change of link; (**b**) the comparison result of link angle value and sensor data at low speed (0.89 degrees/sec); (**c**) the comparison result of link angle value and sensor data at middle speed (3.96 degrees/sec).

In order to check whether the sensor data can be acquired stably when the slope of the link is dynamically changed, the measurement was carried out as follows. As shown in Figure 12a, with a 4 kg load pre-applied, the slope of the link moved in the −30 to 30 degrees angle range. The slope change speed was controlled in two ways—low speed (0.89 degree/sec) and middle speed (3.96 degree/sec)—and repeated twice. The Mz sensor was measured, and the moment value was expressed using a matrix in Equation (21), to observe the change. As in Figure 12b,c, the sensor data responded well to the change in the angle of the link under different speed conditions. The change of angle and moment value of the link could be observed according to the speed change, and the value change became gradual when the inclination direction of the link changes. Through this experiment, the dynamic external force detection performance of the sensor according to link slope change speed was confirmed, and it is expected that the dynamic performance of more diverse sensors can be evaluated through the improvement of the measurement environment.

## 5. Conclusions

In this paper, a link-type three-axis sensor embedded in a cylindrical structure is proposed for effective collision detection between a worker and a collaborative robot. It shows high precision with a simple structure and low cost compared to conventional sensors. The proposed sensor system has several characteristics that are desirable for a high performance force sensor. First, stable external force estimation and collision detection performance were confirmed using linearity sensor output values in an operating environment where simple repetitive tasks occur. Second, the theoretical calculation results, structural analysis results, and external force calculation results through calibration were in good agreement. Through the weight test, the bending moment and torsion moment were able to detect micro external force changes of 0.02 Nm and 0.01 Nm, respectively. Third, the sensor’s excellent output characteristics, repeated durability reliability, and dynamic state detection performance were confirmed. Since the movement of the robot arm can be considered as a quasi-static behavior, it can be expected that there will be no major problems in the future dynamic situation by using excellent sensing performance. Therefore, collisions with workers can be efficiently detected, and can be easily and simply applied in various industrial sites. As a follow-up study, combined with AI models and machine learning technology, we plan to effectively detect external forces even in dynamic situations, which will enable us to more effectively detect various safety problems that may occur in collaborative robot working environments.

## Data Availability

Not applicable.
